# Psychological distress in Aotearoa New Zealand adults with type 1 diabetes

**DOI:** 10.1177/13591053241289189

**Published:** 2024-10-28

**Authors:** Joanna M McClintock, Lynne Chepulis, Tania Blackmore, Sonya Fraser, Ryan G Paul

**Affiliations:** 1 Te Whatu Ora Waikato, New Zealand; 2 Te Huataki Waiora School of Health, University of Waikato, New Zealand

**Keywords:** adults with type 1 diabetes, disordered eating, fear of hypoglycaemia, Indigenous people with diabetes, psychological distress, type 1 diabetes

## Abstract

The psychological burden of type 1 diabetes (T1D) can negatively impact health outcomes. This study evaluates the prevalence of low mood (WHO-5), disordered eating (DEPS-R), diabetes distress (PAID) and fear of hypoglycaemia (HFS-II), in a sample of 250 New Zealand adults (8.4% Māori/91.6% non-Māori; 43.6% female/56.4% male) with T1D using validated tools. Māori and female patients indicated low mood, with lower median WHO-5 scores than non-Māori (*p* = 0.027) and males (*p* = 0.002). Māori were more likely to score in the clinical range on the WHO-5, DEPS-R, PAID and HFS-II (all *p* < 0.05). HbA1c was correlated with emotional well-being (*r_s_* = −0.189), diabetes distress (*r_s_* = 0.223) and disordered eating (*r_s_* = 0.389; all *p* < 0.001) whilst DEPS-R correlated with age (*r_s_* = −0.232) and BMI (*r_s_* = 0.343; both *p* ≤ 0.001). Thus, diabetes-related psychological distress is common in New Zealand adults with T1D, particularly for Māori, females and those with elevated HbA1c levels.

## Introduction

The role of psychological factors in type 1 diabetes (T1D) is well-recognised (Sturt et al., 2015). Due to the intensity of lifelong daily management (Ministry of Health, 2014) low mood and depression as well as distress related to diabetes specific factors including glucose levels (specifically fear of hypoglycaemia), and risk of complications have been identified as having direct relationships with poorer glycaemic control (Snoek et al., 2011; Speight et al., 2012). Due to the focus on food intake and a person’s physical body, disordered eating is an additional psychological concern in adults with T1D and is also positively correlated with HbA1c (Watt et al., 2022; Wisting et al., 2018). There is also likely a reciprocal relationship, where suboptimal glycaemic control contributes to psychological distress, and these emotional and cognitive behavioural responses can then result in further worsening glycaemic control (Shaban et al., 2009).

Although not as widely studied as in the child and youth populations (Bachmeier et al., 2020; d’Emden et al., 2017; McClintock et al., 2022; Nip et al., 2019; Speight et al., 2015) diabetes distress appears to be common in adults with T1D, more specifically in women, those with more suboptimal control or burden of complications (Dennick et al., 2015; Fisher et al., 2016; Snell, 2011; van Duinkerken et al., 2020). However, currently there is a dearth of data exploring these variables in New Zealand as well as in indigenous peoples with T1D in general.

Ethnic differences in rates of psychological distress are also well described in youth and emerging adults with T1D (Nip et al., 2019; Snell, 2011; Watt et al., 2022) and in adults with type 2 diabetes (Agarwal et al., 2020). However, the impact of ethnicity on diabetes distress in adults with T1D is less well known (Özcan et al., 2018). This is of particular interest for Aotearoa New Zealand, as Māori, the indigenous people of New Zealand, have higher rates of general psychological concerns than non-Māori, which is understood to be largely a reflection of social determinants of health (Russell, 2018). These inequities continue to be a challenge for healthcare in Aotearoa New Zealand and there is a long way to go to meet the psychological needs of Māori, and to meet the recommendations of national and international diabetes care guidelines (Ministry of Health, 2014; National Institute of Health and Care Excellence, 2016; Robinson et al., 2018; Young-Hyman et al., 2016).

The importance of screening for psychological distress related to diabetes alongside mental health conditions is highlighted in diabetes guidelines around the world (Hagger et al., 2016; Pouwer and Speight, 2017; Robinson et al., 2018; Young-Hyman et al., 2016). Particularly as psychological factors represent a significant barrier to diabetes management, increasing the risk of physical and mental health complications and ultimately, contributing to increased morbidity and mortality (National Institute for Health and Care Excellence, 2016). There is also likely a reciprocal relationship, where suboptimal glycaemic control contributes to psychological distress, and these emotional and cognitive behavioural responses can then result in further worsening glycaemic control (Shaban et al., 2009).

As the breadth of this psychological distress in adults with T1D is largely unexplored in Aotearoa New Zealand and elsewhere, this study aims to document the prevalence of psychological distress in a sample of adult New Zealanders living with T1D. An additional objective was to confirm the hypothesis that psychological distress associates positively with diabetes-related clinical indicators. Furthermore, this study aimed to determine if the inequities seen in physical diabetes health outcomes for Māori are also evident in measures that assess the psychological aspects of diabetes.

## Methods

This study involved a survey of adult T1D patients (aged ≥20 years), along with collection of patient-specific clinical information. All patients had been diagnosed at least 1 year prior to the survey/clinic visit (either diagnosed by an endocrinologist or had positive anti-GAD and/or anti-IA2 antibodies), and all had severe endogenous insulin deficiency (defined as undetectable C-peptide in the absence of hypoglycaemia).

Patients were recruited as they attended the Waikato Regional Diabetes Service (WRDS) between January 2019 and January 2020 for their annual specialist clinical appointment (with Dr Ryan Paul) All adult patients were invited to complete the questionnaire prior to their clinic appointment, though 11 patients did not complete the questionnaire due to either language difficulties (*n* = 7) or visual impairment (*n* = 4).

The questionnaire included 82 questions/statements to assess a number of psychological measures (see below). Completed questionnaires were labelled with the patient national health index (NHI) number to allow for later identification and linkage to clinical data.

Clinical data was obtained via electronic records at the WRDS. These data included patient age (at time of appointment), gender, ethnicity, most recent HbA1c, any co-diagnosis of coeliac disease (Y/N), duration of T1D, C peptide levels, body mass index (BMI; collected from clinic records) and mode of insulin delivery (continuous subcutaneous insulin infusion; CSII vs multiple daily injections; MDI) and social deprivation data (deprivation is based on region of domicile is coded from 1 to 10, where 1 represents the areas with the least deprivation and 10 the areas with the most deprivation (NZDep2018; Atkinson et al., 2019)). This index also contains census indicators such as income, home ownership, financial support, qualifications, and employment, transport and internet access. Ethnicity was categorised as Māori or non-Māori based on the recorded self-identified ethnicity (using the standard ethnicity census question).

Ethical approval was obtained from the New Zealand Health and Disabilities Ethics Committee: reference 17/NTB/43.

### Psychological measures

The questionnaire was a collation of validated tools and contained questions about the prevalence of low mood, disordered eating, diabetes distress and fear of hypoglycaemia among patients diagnosed with T1D. Emotional well-being was assessed using The World Health Organisation Well-Being Index (WHO-5; Bech, 2004), a positively-worded 5-item scale with responses scored using a 6-point Likert scale ranging from 0 (not present) to 5 (present all of the time). The item scores are summed and multiplied by 4 to provide a percentage score, with low scores suggestive of poor emotional well-being. The clinically significant cut-off score for the WHO-5 is 50 when using this test to screen for depression (Topp et al., 2015), with scores at 50 or below suggestive of low mood.

The Diabetes Eating Problem Survey-Revised (DEPS-R) is a 16-item measure to assess general and diabetes-specific aspects of disordered eating (Markowitz et al., 2010). Responses are scored using a 6-point Likert scale ranging from 0 (never) to 5 (always), with higher scores indicating higher risk of disordered eating. Scores ≥20 on the DEPS-R are clinically significant and considered indicative of notable eating issues (Markowitz et al., 2010). Originally developed for paediatric population, the DEPS-R has been validated for adults with T1D (Wisting et al., 2019).

Diabetes distress was determined by the Problem Areas in Diabetes Scales (PAID; Welch et al., 1997), a 20 item scale where patients indicate the extent to which each item is a problem on a 5-point scale ranging from 0 (not a problem) to 4 (serious problem). Item scores are then summed and multiplied by 1.25, with scores ≥40 suggestive of emotional distress (Snoek et al., 2011).

Fear of hypoglycaemia was assessed by the Hypoglycaemia Fear Survey (HFS-II; Gonder-Frederick et al., 2011), a 32-item self-report measure assessing both *behaviours* used to avoid hypoglycaemia and its negative consequences and *worry* about different anxiety-provoking aspects of hypoglycaemia. Responses are scored according to a 5-point Likert scale ranging from 0 (never) to 4 (almost always), with higher scores suggestive of fear of hypoglycaemia. Due to the complex interplay between actual risk of hypoglycaemia and fear of hypoglycaemia, no clinical cut-off is available. However, Gonder-Frederick (2019, personal communication) recommends using one standard deviation above (or below) the group mean to calculate clinically significant scores. For this sample, scores ≥23 were considered clinically significant for the behaviour scale and scores ≥22 for the worry scale for the HFS-II.

### Statistical methods

Data were summarised using frequency distributions and descriptive analysis. Due to non-normally distributed data, Independent Sample Median tests for non-parametric data and Kruskal-Wallis were used to compare the median test scores for each psychological scale, analysed by ethnicity, gender and type of insulin therapy (CSII vs MDI). Spearman’s correlation coefficient was used to examine the relationship between test scores, age and HbA1c. For all psychological questionnaires, a sub-group analysis of clinically significant cut-off scores yielded small sample sizes, so Fishers Exact tests were used to compare the proportion of patients who scored above and below the clinically significant cut-off scores for each scale. All statistical analyses were performed using SPSS version 27 (IBM Corporation, New York, NY, USA). All tests for significance were two-tailed with *p* < 0.05 considered a statistically significant result.

## Results

The characteristics of all 250 patients are shown in Table 1. Overall, 91.6% were non-Māori, 56.4% were male and 70.8% were MDI users. Only 7.2% of all patients had coeliac disease. The median duration of diabetes was 19.0 years and over half lived with significant social deprivation (SES 7–10).

**Table 1.** Characteristics of adult Waikato diabetes clinic patients (*n* = 250).

Factors	*n* = 250	%
Ethnicity		
Māori	21	8.4
Non-Māori	229	91.6
Gender		
Male	141	56.4
Female	109	43.6
C peptide		
<30 pmol/L	150	60.0
>30 pmol/L	66	26.4
Unknown	34	13.6
Insulin therapy		
MDI	177	70.8
CSII	73	29.2
Coeliac disease		
No	213	85.2
Yes	18	7.2
Unknown	19	7.6
NZDep		
1–2	33	13.9
3–4	29	12.2
5–6	54	22.8
7–8	75	31.6
9–10	57	24.1
Unknown	2	0.8
Age (continuous), Median (Q1, Q3)	40.3 (26.2, 56.8)	
Most recent HbA1c (mmol/mol), Median (Q1, Q3)	67.0 (60.0, 77.0)	
Duration of diabetes, Median (Q1, Q3)	19.0 (10.8, 31.0)	
BMI, Median (Q1, Q3)	26.9 (23.9, 30.9)	

Table 2 provides a summary of the psychological test scores. As Table 2 demonstrates, Māori patients and female patients were significantly more likely to report higher levels of psychological distress compared to non-Māori patients (across all five domains) and male patients (four domains, excluding the HFS-II B), respectively; however, no differences were observed with regard to presence/absence of coeliac disease, C-peptide level, mode of insulin therapy or deprivation quintile. Of concern, median diabetes distress scores were observed to be two and a half times higher in Māori versus non-Māori patients (31.3 vs 12.5) though the differences between Māori and non-Māori for other domains was considerably smaller.

**Table 2.** Median test scores by ethnicity, gender, C-Peptide result, insulin therapy, coeliac disease and deprivation quintile.

	*n*	Emotional well-being (WHO-5)*		Disordered eating (DEPS-R)		Diabetes specific distress (PAID)		Fear of hypoglycaemia (HFS-B; Behaviour)		Fear of hypoglycaemia (HFS-W; Worry)	
Variable		Median score (Q1, Q3)	*p*	Median score (Q1, Q3)	*p*	Median score (Q1, Q3)	*p*	Median score (Q1, Q3)	*p*	Median score (Q1, Q3)	*p*
Ethnicity											
Māori	21	56.0 (40.0, 68.0)	**0.027**	20.0 (12.3, 28.0)	**0.015**	31.3, (21.3, 46.3)	**<0.001**	18.0 (11.0, 26.0)	**0.044**	12.0 (9.0, 18.0)	**0.048**
Non-Māori	229	68.0 (52.0, 80.0)		12.0 (7.0, 18.0)		12.5 (5.0, 28.8)		13.0 (8.0, 18.0)		10.0 (5.0, 19.0)	
Gender											
Male	141	72.0 (56.0, 80.0)	**0.002**	11.0 (7.0, 17.0)	**0.048**	12.5 (3.8, 28.8)	**0.002**	14.0 (8.0, 18.0)	0.219	8.0 (4.0, 14.5)	**0.026**
Female	109	60.0 (50.0, 76.0)		14.0 (9.0, 19.0)		18.8 (7.5, 30.0)		13.0 (8.0, 19.0)		12.0 (7.0, 21.5)	
C-Peptide											
<30 pmol/L	150	68.0 (52.0, 80.0)	0.497	12.0 (7.8, 18.0)	0.104	15.0 (6.3, 26.3)	0.263	13.0 (8.0, 18.0)	0.805	10.0 (5.0, 17.0)	0.361
>30 pmol/L	66	68.0 (56.0, 80.0)		12.0 (6.0, 18.0)		11.9 (3.8, 31.3)		14.0 (7.8, 19.0)		8.5 (4.5, 19.3)	
Unknown	34	64.0 (52.0, 76.0)		15.0 (10.0, 20.0)		20.0 (5.9, 37.5)		12.8 (8.8, 18.8)		13.5 (6.8, 21.0)	
Insulin therapy											
MDI	177	68.0 (52.0, 80.0)	0.592	13.0 (7.0, 19.0)	0.286	13.8 (4.8, 30.6)	0.493	13.0 (7.5, 19.5)	0.479	10.0 (4.5, 17.0)	0.963
CSII	73	64.0 (50.0, 78.0)		12.0 (8.0, 17.5)		16.3 (8.1, 28.1)		12.5 (9.3, 16.5)		10.0 (5.0, 20.0)	
Coeliac disease											
No	213	64.0 (52.0, 80.0)	0.368	12.0 (8.0, 19.0)	0.202	13.8, (6.3, 28.8)	0.114	13.0(8.0, 18.0)	0.768	10.0 (5.0, 19.0)	0.530
Yes	18	72.0 (55.0, 88.0)		10.5 (6.0, 13.0)		8.8 (5.6, 29.5)		15.0 (8.0, 22.5)		9.0 (4.0, 20.3)	
Unknown	19	68.0 (52.0, 84.0)		16.0 (8.0, 19.0)		23.8 (10.0, 41.3)		15.0 (4.0, 21.0)		9.0 (2.0, 18.0)	
NZDep score											
1–2	33	76.0 (62.0, 82.0)	0.225	12.0 (8.0, 20.0)	0.958	12.5 (5.6, 27.5)	0.776	14.0 (10.5, 18.0)	0.189	7.0 (5.0, 18.0)	0.951
3–4	29	64.0 (52.0, 80.0)		14.0 (6.0, 23.0)		15.0 (10.6, 28.8)		10.0 (7.5, 15.5)		9.0 (5.0, 20.3)	
5–6	54	68.0 (59.0, 76.0)		13.0 (7.0, 19.0)		12.5 (3.8, 30.3)		12.0 (7.8, 18.0)		10.0 (4.0, 17.0)	
7–8	75	62.0 (52.0, 76.0)		12.0 (9.0, 17.0)		16.3 (6.3, 30.0)		13.0 (7.0, 18.0)		10.0 (5.0, 21.0)	
9–10	57	64.0 (50.0, 80.0)		11.0 (6.0, 17.5)		17.5 (4.4, 30.3)		16.0 (9.0, 24.0)		12.0 (5.0, 19.5)	
Unknown	2	66.0 (52.0)		13.5 (13.0)		7.5 (2.5)		12.0 (11.0)		8.0 (5.0)	

* Note that scores on the WHO-5 proceed in a reverse direction to the other scales (low scores on the WHO-5 indicate low mood, high scores on the DEPS-R, PAID and HFS-II indicate high psychological distress).

Bolded P values are those that are statistically significant (i.e. *P* < 0.05).

**Figure 1.**
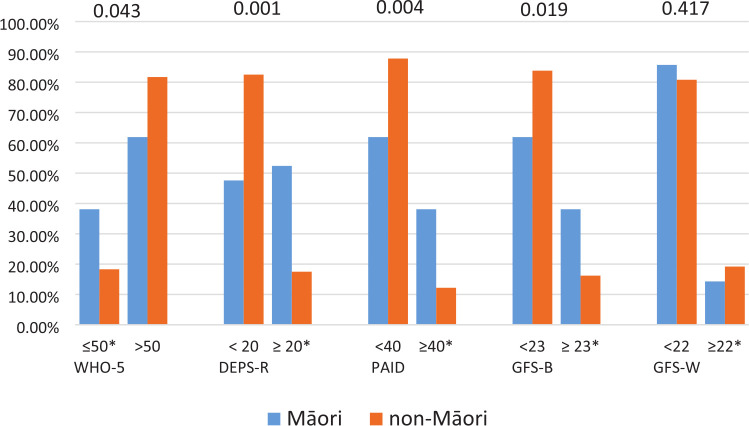
Proportion of patients (Māori vs non-Māori) with Type 1 diabetes who have clinically relevant psychological scores (denoted with a * [≤50 for the WHO-5 and ≥20, 40, 23 and 22, respectively for the DEPS-R, PAID, GFS-B and GFS-W]).

Similarly, when reviewing the proportions of patients who scored above and below the clinical cut-offs for each of these domains Māori patients were significantly more likely than non-Māori patients to clinically score high for low mood (≤50; WHO-5) or to clinically score high for disordered eating (≥20; DEPS-R), diabetes specific distress (≥40; PAID) and fear of hypoglycaemia-behaviour (≥23; HFS-II B; Figure 1). No other numerically significant differences were observed for other variables or domains, other than for females who were more likely than males to have a clinically high score for fear of hypoglycaemia-worry (24.8% of females vs 14.2% of males with HFS-W ≥22; *p* = 0.036). Overall, the proportion of patients with clinically significant scores was 20.0% (WHO-5), 20.4% (DEPS-R), 14.4% (PAID), 18.0% (GFS-B) and 18.8% (GFS-W).

Emotional well-being scores (WHO-5) were inversely correlated with HbA1c (*r_s_* = −0.189, *p* = 0.003) indicating that patients were more likely to score with low mood as median HbA1c levels increased. Both DEPS-R (disordered eating) and PAID (diabetes specific distress) scores were positively correlated with both HbA1c and BMI (all <0.001) suggesting that both weight and blood glucose levels influence eating behaviours and distress. However, DEPS-R scores were inversely correlated with age (*r_s_* = −0.232; *p* < 0.001). No other associations between psychological domains and T1D variables were observed (see Table 3).

**Table 3.** The correlation between each psychological scale and age, HbA1c, BMI, C-Peptide result and deprivation score.

Variable		Age	HBA1c	BMI	C-Peptide	Deprivation score	WHO-5	DEPS-R	PAID	HFS-II (B)	HFS-II (W)
Age	Correlation (*r_s_*)	1.000	−0.239	0.112	−0.066	−0.083	0.075	−0.232	−0.098	0.020	0.045
	*p* Value	.	<0.001*	0.082	0.597	0.191	0.239	<0.001*	0.123	0.748	0.479
HBA1c	Correlation (*r_s_*)	−0.239	1.000	0.022	−0.030	0.126	−0.189	0.389	0.233	0.121	0.106
	*p* Value	<0.001*	.	0.732	0.809	0.047*	0.003*	<0.001*	<0.001*	0.057	0.094
BMI	Correlation (*r_s_*)	0.112	0.022	1.000	0.066	−0.074	−0.025	0.343	0.141	0.003*	0.045
	*p* Value	0.082	0.732	.	0.598	0.252	0.697	<0.001*	0.029*	0.969	0.489
C-Peptide	Correlation (*r_s_*)	−0.066	−0.030	0.066	1.000	−0.022	−0.087	0.091	0.112	0.121	0.073
	*p* Value	0.597	0.809	0.598	.	0.860	0.490	0.467	0.371	0.333	0.560
Deprivation score	Correlation (*r_s_*)	−0.083	0.126	−0.074	−0.022	1.000	−0.123	−0.038	0.014	0.075	0.053
	*p* Value	0.191	0.047*	0.252	0.860	.	0.053	0.548	0.822	0.239	0.407
WHO-5	Correlation (*r_s_*)	0.075	−0.189	−0.025	−0.087	−0.123	1.000	−0.323	−0.427	−0.149	−0.260
	*p* Value	0.239	0.003*	0.697	0.490	0.053	.	<0.001*	<0.001*	0.018*	<0.001*
DEPS-R	Correlation (*r_s_*)	−0.232	0.389	0.343	0.091	−0.038	−0.323	1.000	0.576	0.185	0.318
	*p* Value	<0.001*	<0.001*	<0.001*	0.467	0.548	<0.001*	.	<0.001*	0.003*	<0.001*
PAID	Correlation (*r_s_*)	−0.098	0.233	0.141	0.112	0.014	−0.427	0.576	1.000	0.362	0.591
	*p* Value	0.123	<0.001*	0.029*	0.371	0.822	<0.001*	<0.001*	.	<0.001*	<0.001*
HFS-II (B)	Correlation (*r_s_*)	0.020	0.121	0.003	0.121	0.075	−0.149	0.185	0.362	1.000	0.507
	*p* Value	0.748	0.057	0.969	0.333	0.239	0.018*	0.003*	<0.001*	.	<0.001*
HFS-II (W)	Correlation (*r_s_*)	0.045	0.106	0.045	0.073	0.053	−0.260	0.318	0.591	0.507	1.000
	*p* Value	0.479	0.094	0.489	0.560	0.407	<0.001*	<0.001*	<0.001*	<0.001*	.

* Significant at 0.05.Bolded values are statistically significant *P* values (*P* < 0.05)

## Discussion

This cross-sectional study assessed the psychological factors in 250 adults with T1D receiving specialist care in the Waikato region of Aotearoa New Zealand. Approximately one in five patients experienced some degree of psychological distress, reporting clinically significant scores. However, we note that these data were collected just prior to the COVID pandemic, and the proportion of patients experiencing distress may well have increased as a result since then.

Of particular concern for Aotearoa New Zealand is the finding that Māori had statistically significantly greater distress in all psychological domains than non-Māori except for the behaviour subscale of the Fear of Hypoglycaemia Scale. Unfortunately, ethnic disparities have been identified in previous international research where indigenous or minority groups experience higher levels of psychological distress (Watt et al., 2022) including diabetes distress (Agarwal et al., 2020). Importantly, rates of low emotional wellbeing for Māori in our study were 50% higher than the general population (Statistics New Zealand, 2018) and double those in youth and emerging adults with T1D in our service (McClintock et al., 2022), confirming the impact of T1D on low mood with advancing age (van Duinkerken et al., 2020). This research reinforces the inequities seen between Māori and non-Māori with T1D in other studies (Chepulis et al., 2021; Hill et al., 2017) and aligns with previous national research where Māori were identified to have higher rates of disordered eating than non-Māori (Russell, 2018). Such disparity needs to urgently be addressed and culturally-appropriate programmes are required to mitigate this distress.

Somewhat surprisingly no significant differences in psychological distress were identified between those on CSII and MDI insulin treatment; however, this is similar to the findings of the earlier research with youth and young adults (McClintock et al., 2022) and also Nagel et al. (2021). Additionally, this could be an artifact of the selection process for insulin pumps within Aotearoa New Zealand, where criteria is centred around HbA1c (Pharmac, 2016) and is not patient-driven. We also note that there were no differences in the psychological profile between those with coeliac disease and those without. Although no specific questions related to coeliac disease distress were included, this suggests that there is not a cumulative or double burden effect given the relatively high prevalence of coeliac disease in this population group (Elfström et al., 2014). It is also unexpected that endogenous production of insulin appeared to have no effect on rates of diabetes distress, given the typically greater burden of complications and glycaemia. However, we note that we did not have complete C-peptide data to explore this in any detail and further research understanding the relationship between endogenous insulin production, glycaemic control and psychological distress is warranted.

As this was a cross-sectional study, causation cannot be assumed. Other considerations for this study are that the patients included in this study were all under the care of one of the endocrinologists within the service [Dr Ryan Paul], which has the potential of a selection bias. Further, this study only involved those who attended the clinic in person and therefore will not be reflective of those who are disengaged from specialist diabetes health care. For this reason, the results may underrepresent the levels of psychological distress being experienced by all of those in the area living with T1D. It is also important to note that while we have observed significant differences in the level of psychological distress observed by Māori versus non-Māori participants, the number of Māori included in our study was small (21 of 250 patients). However, our dataset did include all patients who were seen in the clinic during the 12-month study period and is primarily reflective of the lower prevalence and lower median age of T1D seen in Māori populations (Wu et al., 2023) and the fact that all other ethnicities (Asian, Pacific, Middle Eastern, etc.) were collectively grouped together as ‘non-Māori’.

Lastly, while our study used validated survey tools, we note that the questionnaires used were not developed for an Aotearoa New Zealand population, although they have been used with other age ranges, have good face-validity and were reviewed by the multidisciplinary team. Additionally, how applicable they are to an indigenous population needs to be acknowledged as well as recognising that social determinants of health are likely influencing the overall psychological state of adults living in Aotearoa New Zealand. Data on the use of glucose monitoring devices was also not collected which may influence distress levels. However, these devices are not funded in Aotearoa at this time and the uptake is not as high as in countries where they are funded.

In conclusion, in adult New Zealanders living with T1D, the rates of psychological distress are concerning, particularly for females and for Māori. These results provide an objective rationale for screening of psychological distress to be routine in diabetes care in Aotearoa, aligning with international guidelines. Only through screening can psychological needs be identified allowing for targeted interventions by all members of the multidisciplinary team. These interventions can be focused on the area of specific need for that person, including but not limited to education, validation and emotion-focused coping (Hessler et al., 2020). These findings also provide evidence for why diabetes-focused psychology services should be a core component of diabetes clinics. The inclusion of screening and psychological care also emphasises the importance of taking a holistic approach to diabetes management, which is consistent with a Māori world view and will improve equity for Māori.

Lastly, further research is needed in Aotearoa New Zealand. This study is an initial step towards developing normative data for adult New Zealanders living with type 1 diabetes, which can be further developed. It is also important for future research, both nationally and internationally, to gain a better understanding of the psychological profile of indigenous people living with T1D; to improve both physical and psychological outcomes as well as reduce inequities of care.
